# Attenuated RANKL-induced cytotoxicity by *Portulaca oleracea* ethanol extract enhances RANKL-mediated osteoclastogenesis

**DOI:** 10.1186/s12906-015-0770-9

**Published:** 2015-07-14

**Authors:** Munkhsoyol Erkhembaatar, Eun-Joo Choi, Hak-Yong Lee, Choong Hun Lee, Young-Rae Lee, Min Seuk Kim

**Affiliations:** Department of Oral Physiology, and Institute of Biomaterial-Implant, College of Dentistry, Wonkwang University, Iksan, Jeonbuk 570-749 Republic of Korea; Department of Oral and Maxillofacial Surgery, College of Dentistry, Wonkwang University, Iksan, Jeonbuk 570-749 Republic of Korea; Huvet Inc., 160 Yakchon-ro, Iksan, Jeonbuk 570-979 Republic of Korea; Microelectronics and Display Technology, Next Generation Industrial Radiation Technology RIC, Center for PV Human Resource Development, Wonkwang University, Iksan, 570-749 Republic of Korea; Center for Metabolic Function Regulation (CMFR), Wonkwang University School of Medicine, Iksan, Jeonbuk 570-749 Republic of Korea; Department of Oral Biochemistry, and Institute of Biomaterial-Implant, College of Dentistry, Wonkwang University, 344-2 Shinyong-dong, Iksan, 570-749 Republic of Korea

**Keywords:** *Portulaca oleracea*, Receptor activator of NF-κB ligand, Bone marrow-derived macrophage, Osteoclastogenesis

## Abstract

**Background:**

*Portulaca oleracea* (PO) has been widely used as traditional medicine because of its pharmacological activities. However, the effects of PO on osteoclasts that modulate bone homeostasis are still elusive.

**Methods:**

In this study, we examined the effects of PO ethanol extract (POEE) on receptor activator of nuclear factor-κB ligand (RANKL)-mediated Ca^2+^ mobilization, nuclear factor of activated T-cell c1 (NFATc1) amplification, tartrate-resistant acid phosphatase-positive (TRAP+) multinucleated cell (MNC) formation, and cytotoxicity.

**Results:**

Our results demonstrated that POEE suppressed RANKL-induced Ca^2+^ oscillations by inhibition of Ca^2+^ release from internal Ca^2+^ stores, resulting in reduction of NFATc1 amplification. Notably, POEE attenuated RANKL-mediated cytotoxicity and cleavage of polyadenosine 5′-diphosphate-ribose polymerase (PARP), resulted in enhanced formation of TRAP+ MNCs.

**Conclusions:**

These results present *in vitro* effects of POEE on RANKL-mediated osteoclastogenesis and suggest the possible use of PO in treating bone disorders, such as osteopetrosis.

## Background

*Portulaca oleracea* (PO), also known as verdolaga and pigweed, has been widely used not only as food, but also as traditional medicine treating insect bites, bacillary dysentery, diarrhea, and hemorrhoids. The chemical constituents of PO have been repeatedly reported to have diverse pharmacological activities. For instance, polysaccharides and betacyanins isolated from PO have antifatigue effects and improve cognition deficits in mice, respectively [1, 2]. Importantly, PO ethanol extract (POEE) is known to have protective effects against ultraviolet-induced apoptosis in keratinocytes and fibroblasts [3], whereas POEE elicits cytotoxicity in cancer cells [4]. These accumulated evidences suggest the multiple effects of PO, which plays different roles dependent on cell type.

Bone is constantly being remodeled by the delicate balance between the activities of osteoblasts, which are in charge of bone mineralization, and osteoclasts, which resorb bone matrix. In the process of bone remodeling, receptor activator of nuclear factor-κB ligand (RANKL), a key molecule expressed in osteoblasts, mediates osteoclastogenesis resulting in breakdown of bone. Contact between RANKL and receptor activator of nuclear factor-κB (RANK), expressed on osteoclast precursor cells, mediates differentiation-related signals through nuclear factor of activated T-cell c1 (NFATc1) activation [5]. Notably, repeated reports have clearly showed that RANKL-induced free cytosolic Ca^2+^ ([Ca^2+^]_i_) oscillations modulate NFATc1 activity [5–7]. Generation of long-lasting [Ca^2+^]_i_ oscillations sequentially activates Ca^2+^/calmodulin-dependent protein kinase, calcineurin, and NFATc1. Activated NFATc1 accumulates inside the cell nucleus, eliciting the induction of gene expression necessary for osteoclastogenesis. On the other hand, RANKL is also known to inhibit cell proliferation and induce apoptosis through a tumor necrosis factor receptor-associated factor 6 (TRAF6)-dependent but NF-κB-independent mechanism [8]. However, the signal pathways underlying these opposing effects of RANKL-RANK contact are less well understood.

In this study, we report *in vitro* effects of POEE, which attenuates RANKL-induced cytotoxicity and enhances RANKL-mediated osteoclastogenesis. Our findings may suggests the possible use of PO to modulate the activity of osteoclast.

## Methods

### Cell culture and reagents

BMMs isolation was conducted in accordance with the protocols approved by the Institutional Animal Care and Use Committee of Wonkwang University (committee member: Sung Yeon Kim, Jungkee Kwon, Hong Geun Oh, Hong-Seob So, Okjin Kim, Chun-Soo Ko). Primary BMMs were cultured in alpha-modified minimum essential medium (α-MEM; Sigma-Aldrich, MO, USA) supplemented with 10 % fetal bovine serum (FBS) and 30 ng/mL macrophage colony-stimulating factor (M-CSF) and incubated in 5 % CO_2_. PO collected at a local farm (Duhak-dong, Jecheon-si, Chungcheongbuk-do, Republic of Korea) was purchased from the University Oriental Herbal Drugstore (Iksan, Republic of Korea), and it was authenticated by Oriental pharmacologist Jang-Ho Ko (Huvet Inc., Iksan, Republic of Korea). A voucher specimen has not been deposited in a public herbarium. Dried PO was ground and extracted with ethanol for 3 h at 70 °C. After filtering, the precipitate was collected and vaccum-dried at 78 °C and then used for each experiment. Soluble recombinant mouse RANKL and M-CSF were purchased from KOMA Biotech (Seoul, Korea). Cyclopiazonic acid (CPA) and adenosine triphosphate (ATP) were obtained from TOCRIS Bioscience (Bristol, US) and Sigma Aldrich (MO, USA), respectively. Antibodies against polyadenosine 5′-diphosphate-ribose polymerase (PARP), NFATc1, and β-actin were purchased from Cell Signaling Technology (MA, USA), Santa Cruz Biotechnology (TX, USA), and Sigma Aldrich (MO, USA), respectively. Fura-2-acetoxymethyl ester (Fura-2 AM) was obtained from TEFLabs (TX, USA).

### *In vitro* osteoclast formation

Murine BMMs were prepared from the femur and tibia of 4- to 6-week-old mice, as previously described [7]. Briefly, bone marrow was flushed out with culture medium (α-MEM) and collected. After removal of red blood cells, whole marrow cells were plated on uncoated Petri dishes in the presence of M-CSF (10 ng/mL). The following day, the BMMs were collected and then seeded on designated culture dishes for each experiment. Henceforth, M-CSF was supplemented in the culture medium at a concentration of 30 ng/mL. To generate osteoclasts, the BMMs were treated with RANKL (50 ng/mL) for the indicated time. To evaluate the formation of multi-nucleated cells (MNCs) and tartrate-resistant acid phosphatase (TRAP) activity, BMMs were plated on 24-well culture dishes at a density of 1.2 × 10^5^ cells per well. Cytochemical staining for TRAP expression was performed using a leukocyte acid phosphate assay kit (Sigma-Aldrich, MO, USA), following the manufacturer’s procedures. For measurement of total TRAP activity, p-nitrophenyl phosphate (Sigma-Aldrich, MO, USA) substrate was added to the culture medium containing whole lysates of BMMs. Optical density was measured at an absorbance of 405 nm.

### Cytotoxicity measurement

Isolated BMMs, as described in “*In vitro* osteoclast formation,” were plated in 96-well plates at a density of 1 × 10^4^ cells per well. RANKL and POEE were then added to each well at the indicated concentrations. Following incubation with POEE for the indicated length of time, cytotoxicity was determined using the Vybrant cytotoxicity assay kit (Sigma Aldrich, MO, USA), following the manufacturer’s procedure. Optical density was measured using a microplate reader at 530/590 nm (Ex/Em).

### Western blot analysis

Isolated BMMs were plated on 60-mm dishes at a density of 1 × 10^6^ cells. Following incubation under the stated conditions, each sample was lysed in radioimmunoprecipitation assay (RIPA) buffer (25 mM Tris-HCl pH 7.6, 150 mM NaCl, 1 % NP-40, 1 % sodium deoxycholate, 0.1 % SDS) containing protease inhibitors, and the collected whole cell lysates were cleared by centrifugation at 14,000 × *g* for 10 min at 4 °C. Proteins in the total lysates were separated by size using sodium dodecyl sulfate polyacrylamide gel electrophoresis (SDS-PAGE) and transferred onto polyvinylidene difluoride (PVDF) membranes. Membranes were incubated with the indicated antibodies overnight, and immunoreactive proteins were detected using an electrochemiluminescent (ECL) detection system on the following day.

### [Ca^2+^]_i_ measurement

[Ca^2+^]_i_ was determined using the Ca^2+^-sensitive fluorescent dye Fura-2, as described previously [9]. Briefly, isolated BMMs were plated on cover glass at approximately 80 % confluence (6 × 10^5^ cells per 35-mm dish) and cultured in α-MEM medium. After stimulation with RANKL for 2 d, cells plated on cover glass were used as samples for [Ca^2+^]_i_ measurement. The cells on a cover glass were transferred to HEPES buffer containing 10 mmol/L HEPES, 140 mmol/L NaCl, 5 mmol/L KCl, 1 mmol/L MgCl_2_, 1 mmol/L CaCl_2_, and 10 mmol/L glucose, adjusted to pH 7.4 and 310 mOsm, and then loaded with Fura-2 fluorescent Ca^2+^ indicator for 50 min at RT and placed in a chamber connected to a perfusion system. Cells were briefly washed out with regular HEPES buffer and continuously perfused with HEPES buffer without RANKL and M-CSF. Each of the indicated chemicals was diluted in HEPES buffer or Ca^2+^-free HEPES buffer (10 mmol/L HEPES, 140 mmol/L NaCl, 5 mmol/L KCl, 1 mmol/L MgCl2, 1 mmol/L EGTA, and 10 mmol/L glucose, adjusted to pH 7.4 and 310 mOsm) and perfused for the designated length of time. Under continuous perfusion with regular HEPES buffer (37 °C), intracellular fluorescence was excited at dual wavelengths (340 and 380 nm) and emitted fluorescence (510 nm) was captured using a charge-coupled device (CCD) camera. Captured images were digitized and analyzed using MetaFluor software, with data expressed as the ratio of fluorescence intensities (F_340_/F_380_).

### Statistical analysis

Results were analyzed using Student’s 2-tailed *t*-test. Data are presented as mean ± SEM of the stated number of observations obtained from the indicated number of independent experiments. P-values less than 0.05 were considered statistically significant (*P < 0.05, **P < 0.01).

## Results and discussion

### POEE acutely abolished RANKL-induced [Ca^2+^]_i_ oscillations

**Fig. 1:**
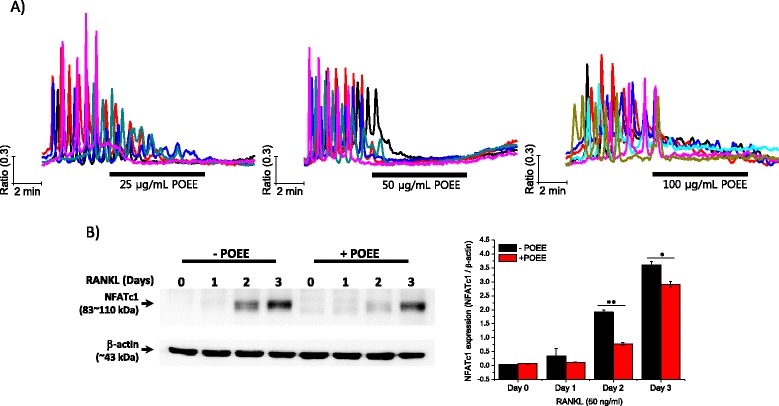
Effects of Portulaca oleracea ethanol extract (POEE) on receptor activator of NF-κB ligand (RANKL)-induced free cytosolic Ca^2+^ ([Ca^2+^]_i_) oscillations and nuclear factor of activated T-cell c1 (NFATc1) amplification. **a** Isolated bone marrow-derived macrophages (BMMs) were plated on cover glass and cultured for 48 h in the presence of RANKL (50 ng/mL). After incubation, [Ca^2+^]_i_ was measured using Fura-2 AM fluorescent dye as described in “Materials and Methods.” To confirm the generation of RANKL-induced [Ca^2+^]_i_ oscillations, cells were initially perfused with regular HEPES buffer, and then acutely treated with 25, 50, and 100 μg/mL of POEE diluted in regular HEPES buffer for the indicated time. Each trace presents the [Ca^2+^]_i_ response of a single cell. **b** Isolated BMMs were treated with RANKL (50 ng/mL) for the indicated time with or without POEE (50 μg/mL). Following incubation, whole proteins were collected and used for determining NFATc1 expression. β-actin was used for loading control. NFATc1 expression is shown as the mean of the ratio (NFATc1/β-actin)

In our previous report, we confirmed that *Glechoma hederacea* ethanol extract elicited a transient increase in [Ca^2+^]_i_ in BMMs, which sequentially suppressed RANKL-mediated [Ca^2+^]_i_ oscillations, NFATc1 activation, and formation of TRAP-positive (TRAP+) MNCs [10]. To validate the physiological roles of POEE in RANKL-mediated osteoclastogenesis, we first examined the acute effects of POEE on RANKL-induced [Ca^2+^]_i_ oscillations. Isolated BMMs were plated on cover glass, and [Ca^2+^]_i_ responses were measured after 2 d of RANKL stimulation. Figure 1a clearly shows that 25 μg/mL of POEE gradually decreased Ca^2+^ peaks, but higher concentrations of POEE (50 and 100 μg/mL) immediately abolished RANKL-induced [Ca^2+^]_i_ oscillations. Notably, abolished RANKL-induced [Ca^2+^]_i_ oscillations were not rescued by removal of POEE. This negative effect of POEE on RANKL-induced [Ca^2+^]_i_ oscillations strongly suggests that POEE may inactivate NFATc1. To examine the effect of POEE on NFATc1 activation, BMMs were simultaneously treated with 50 μg/mL of POEE (the minimum concentration that immediately abolished RANKL-induced [Ca^2+^]_i_ oscillations) and RANKL, and cultured for the indicated time. Figure 1b shows that RANKL-mediated NFATc1 amplification was significantly inhibited by POEE. As reported previously, the presence of [Ca^2+^]_i_ oscillations after 24 h of stimulation with RANKL is critical to activate NFATc1 and trigger the late-stage of osteoclastogenesis [10]. Therefore, we assumed that POEE may negatively regulate RANKL-mediated osteoclastogenesis.

### POEE reduced receptor-mediated Ca^2+^ release from internal Ca^2+^ stores, but not extracellular Ca^2+^ influx via store-operated Ca^2+^ channels

**Fig. 2:**
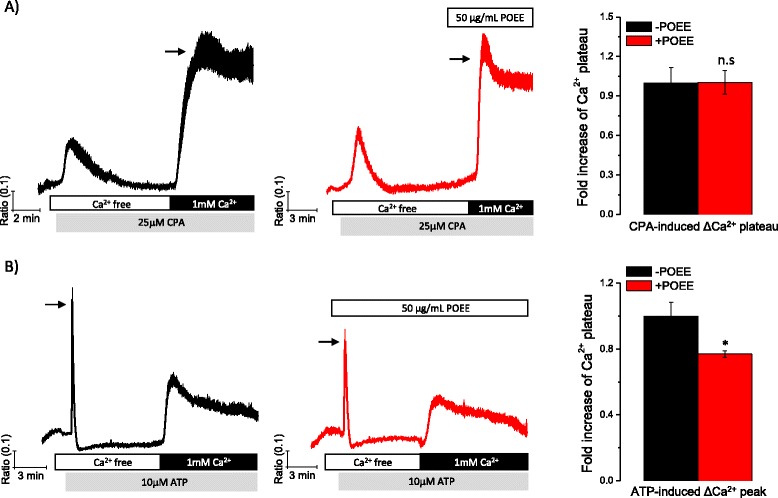
Portulaca oleracea ethanol extract (POEE) reduces receptor-mediated Ca^2+^ release from internal Ca^2+^ stores, but not extracellular Ca^2+^ influx. Isolated bone marrow-derived macrophages (BMMs) were plated on cover glass and cultured for 48 h in the presence of receptor activator of NF-κB ligand (RANKL) (50 ng/mL). After incubation, free cytosolic Ca^2+^ ([Ca^2+^]_i_) was measured. **a** To deplete internal Ca^2+^ stores, extracellular Ca^2+^ was removed by adding 1 mM of EGTA, and BMMs were then treated with 25 μM of cyclopiazonic acid. Following Ca^2+^ store depletion, 1 mM of Ca^2+^ was added back in the presence or absence of POEE. **b** Cells were treated with a low concentration of adenosine triphosphate (ATP) (10 μM) in the absence of extracellular Ca^2+^. Extracellular Ca^2+^ was then restored to determine receptor-mediated extracellular Ca^2+^ influx. ΔCa^2+^ increase (indicated with an arrow) is presented as relative mean fold change

Accumulated evidence has revealed that RANKL-induced [Ca^2+^]_i_ oscillations require orchestration of Ca^2+^ mobilization from internal and external Ca^2+^ stores [7, 11–13]. Especially, our previous work indicated that release of Ca^2+^ from inositol 1,4,5-trisphosphate (IP_3_)-sensitive Ca^2+^ stores and influx of extracellular Ca^2+^ via store-operated Ca^2+^ channels (SOCCs) are both essential for building up RANKL-induced [Ca^2+^]_i_ oscillations [7]. Confirmation of immediate abolishment of RANKL-induced [Ca^2+^]_i_ oscillations led us to further investigate Ca^2+^ mobilization responses following treatment with POEE. To confirm the effect of POEE on Ca^2+^ influx through SOCCs, internal Ca^2+^ stores were depleted by CPA in the absence of extracellular Ca^2+^, and extracellular Ca^2+^ influx through SOCCs was then determined by restoring extracellular Ca^2+^ with or without POEE (Fig. 2a). Results in Fig. 2a show that POEE did not affect the influx of extracellular Ca^2+^ via SOCCs. In a parallel experiment, receptor-mediated Ca^2+^ release from internal Ca^2+^ stores was confirmed by stimulating cells with a low concentration of ATP. It has been reported previously that activation of P2Y receptors with ATP mobilizes internal [Ca^2+^]_i_ from IP_3_-sensitive Ca^2+^ stores in osteoclasts [14]. Considering this, we treated cells with ATP after removal of extracellular Ca^2+^ to evaluate Ca^2+^ release from internal Ca^2+^ stores, followed by the restoration of extracellular Ca^2+^ to confirm receptor activation-mediated extracellular Ca^2+^ influx. Results in Fig. 2b indicate that POEE significantly reduced ATP-mediated Ca^2+^ release from internal Ca^2+^ stores, but not receptor activation-mediated extracellular Ca^2+^ influx. In the present study, we stimulated cells with a low concentration of ATP (10 μM). Interestingly, one study reported that both low and high concentrations of ATP mobilized [Ca^2+^]_i_, but low concentrations of ATP did not induce NF-κB localization into the nucleus, which strongly regulates osteoclast formation [15]. According to this report, the current results suggest that POEE, by reducing ATP-mediated Ca^2+^ release from internal Ca^2+^ stores, may alter NF-κB-independent signal pathways.

### POEE enhanced RANKL-mediated TRAP+ MNC formation

**Fig. 3:**
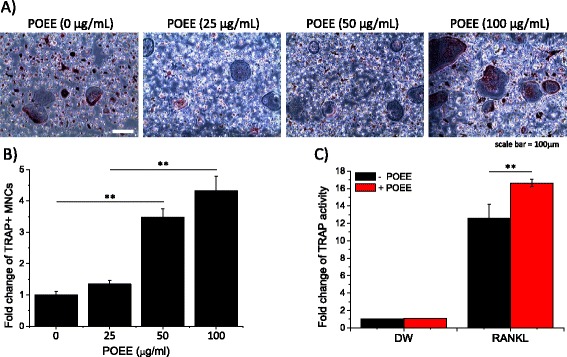
*Portulaca oleracea* ethanol extract (POEE) markedly enhances receptor activator of NF-κB ligand (RANKL)-mediated tartrate-resistant acid phosphatase (TRAP)-positive multinucleated cell (MNC) formation and TRAP activity. **a** and **b** Isolated bone marrow-derived macrophages (BMMs) were simultaneously treated with RANKL (50 ng/mL) and POEE (0, 25, 50, and 100 μg/mL) and then cultured for 4 d. Following incubation, TRAP staining was performed. TRAP-positive MNCs present in each well, identified by the presence of more than 3 nuclei and cell size larger than 100 μm in diameter, were counted. **c** To measure TRAP activity, cells were treated with RANKL (50 ng/mL) and POEE (50 μg/mL) simultaneously. After 4 d of incubation, cells were lysed and total TRAP activity was then measured as described in “Materials and Methods”

Considering the previous findings, Figs. 1 and 2 indicate different possible mechanisms for POEE’s effect on RANKL-mediated osteoclastogenesis. Inhibition of RANKL-induced [Ca^2+^]_i_ oscillations and NFATc1 activation suggests that POEE may suppress cell differentiation into osteoclasts, whereas altered internal Ca^2+^ mobilization in response to a low concentration of ATP suggests that POEE may work in a different way. To confirm this, we performed *in vitro* osteoclast formation assays with and without POEE. Isolated BMMs were stimulated with RANKL and simultaneously treated with POEE in a concentration-dependent manner. Figs. 3a and b show that 50 μg/mL of POEE markedly enhanced RANKL-mediated osteoclastogenesis. Consistent with this finding, 50 μg/mL of POEE significantly increased TRAP activity compared to RANKL-only treated cells (Fig. 3c). These results revealed that, even though POEE suppresses RANKL-induced [Ca^2+^]_i_ oscillations and NFATc1 activation, POEE simultaneously activates other signal pathways resulting in the enhancement of MNC formation. Since Takayanagi et al. suggested that NFATc1 is the master regulator of RANKL-induced osteoclastogenesis [5], this is the first report that suppression of NFATc1 can be overcome by activation of other signal pathways.

### POEE attenuated RANKL-mediated cytotoxicity

**Fig. 4:**
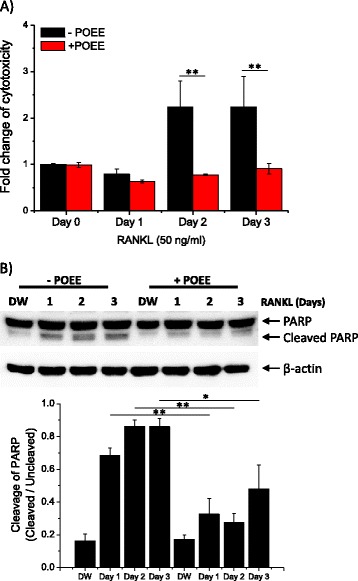
*Portulaca oleracea* ethanol extract (POEE) attenuates receptor activator of NF-κB ligand (RANKL)-mediated cytotoxicity. **a** Bone marrow-derived macrophages (BMMs) in 96-well plates were stimulated with RANKL for the indicated time. Following incubation, glucose-6-phosphate dehydrogenase (G6PD) in the culture medium was measured. The amount of released G6PD is calculated as a percentage of total G6PD, and results are presented as relative mean fold change. **b** BMMs were cultured under the indicated conditions. Polyadenosine 5′-diphosphate-ribose polymerase (PARP) expression and cleavage were evaluated by performing western blot analysis. Summarized data are presented as a ratio of cleaved PARP/uncleaved PARP in lower panel. β-actin was used for loading control

Figures 1 and 2 show that POEE strongly abolished RANKL-induced [Ca^2+^]_i_ oscillations by inhibiting internal Ca^2+^ mobilization, which is known as a critical factor for osteoclastogenesis, whereas Fig. 3 clearly shows that treatment with POEE enhanced TRAP+ MNC formation. These paradoxical results raise questions about the dual effects of POEE on signal pathways mediated by RANKL. Although RANK/RANKL’s prosurvival role through activation of NF-κB is well known [16], Bharti et al. reported that contact between RANK and RANKL can suppress cell proliferation and induce apoptosis by activating a NF-κB-independent and TRAF6-dependent mechanism [8]. According to this report, we hypothesized that POEE may have protective effects against RANKL-mediated cytotoxicity, resulting in the enhancement of TRAP+ MNC formation. To confirm this, *in vitro* osteoclast formation assays were performed with and without POEE. After various incubation periods, cytotoxicity was measured by evaluating the amount of glucose-6-phosphate dehydrogenase (G6PD) released in the culture medium. Figure 4 shows that RANKL by itself induced cytotoxicity after 2 d of incubation, whereas RANKL-mediated cytotoxicity was attenuated by POEE (50 μg/mL). Cleavage of caspase and PARP is associated with cell apoptosis [17]. Based on this, we further confirmed the level of PARP and its cleavage in RANKL-mediated osteoclastogenesis. Compared with results shown in Fig. 4a, a significant decrease in PARP expression and cleavage was observed in POEE-treated cells (Fig. 4b). Accumulated evidences have demonstrated that RANKL stimulation on bone marrow-derived macrophages (BMMs) transmit diverse signal pathways which mediate numerous and various cellular responses, such as cellular motility, membrane fusion, differentiation-related gene expression, and apoptosis [5, 8, 18, 19]. Orchestrated RANKL-mediated signal pathways lastly result in differentiation of osteoclasts.

According to our results and other reports, RANKL is known to not only elicit Ca^2+^ - Ca^2+^/calmodulin-dependent kinase – calcineurin – NFATc1 signal pathway, which induces differentiation-related gene expression [5], but also suppress cell proliferation and induce apoptosis through TRAF6-dependent mechanism [8]. These reports obviously show that these conflicting signal pathways are both involved in osteoclastogenesis. Notably, NFATc1 activation occurs in relatively early stage of osteoclastogenesis, whereas apoptosis is induced in relatively late stage of osteoclastogenesis [8]. Considering these, our data clearly demonstrate that POEE has multiple effects on RANKL-mediated osteoclastogensis. In early stage, POEE suppresses RANKL-induced Ca^2+^ oscillation and NFATc1 expression, whereas POEE attenuates RANKL-mediated cytotoxicity in late stage, resulting in enhanced multi-nucleated cells formation. Taken together, anti-apoptotic function of POEE is more essential and dominant effect on RANKL-mediated osteoclastogenesis.

## Conclusions

Our results demonstrate that POEE has dual and opposite effects on RANKL-mediated osteoclastogenesis. POEE not only inhibits the generation of RANKL-induced [Ca^2+^]_i_ oscillations and NFATc1 activation but also markedly enhances RANKL-mediated osteoclastogenesis by attenuating RANKL-induced cytotoxicity. Our findings suggest that the RANKL-induced cytotoxicity caused by Ca^2+^ release from internal Ca^2+^ store is attenuated by POEE, which resulted in enhanced RANKL-mediated osteoclastogenesis.

## References

[1] Xu Z, Shan Y (2014). Anti-fatigue effects of polysaccharides extracted from Portulaca oleracea L. in mice. Indian J Biochem Biophysics.

[2] Wang CQ, Yang GQ (2010). Betacyanins from Portulaca oleracea L. ameliorate cognition deficits and attenuate oxidative damage induced by D-galactose in the brains of senescent mice. Phytomed.

[3] Lee S, Kim KH, Park C, Lee JS, Kim YH (2014). Portulaca oleracea extracts protect human keratinocytes and fibroblasts from UV-induced apoptosis. Exp Dermatol.

[4] Farshori NN, Al-Sheddi ES, Al-Oqail MM, Musarrat J, Al-Khedhairy AA, Siddiqui MA (2014). Cytotoxicity assessments of Portulaca oleracea and Petroselinum sativum seed extracts on human hepatocellular carcinoma cells (HepG2). Asian Pacific J Cancer Prev.

[5] Takayanagi H, Kim S, Koga T, Nishina H, Isshiki M, Yoshida H, Saiura A, Isobe M, Yokochi T, Inoue J (2002). Induction and activation of the transcription factor NFATc1 (NFAT2) integrate RANKL signaling in terminal differentiation of osteoclasts. Dev Cell.

[6] Koga T, Inui M, Inoue K, Kim S, Suematsu A, Kobayashi E, Iwata T, Ohnishi H, Matozaki T, Kodama T (2004). Costimulatory signals mediated by the ITAM motif cooperate with RANKL for bone homeostasis. Nature.

[7] Kim MS, Yang YM, Son A, Tian YS, Lee SI, Kang SW, Muallem S, Shin DM (2010). RANKL-mediated reactive oxygen species pathway that induces long lasting Ca2+ oscillations essential for osteoclastogenesis. J Biol Chem.

[8] Bharti AC, Takada Y, Shishodia S, Aggarwal BB (2004). Evidence that receptor activator of nuclear factor (NF)-kappaB ligand can suppress cell proliferation and induce apoptosis through activation of a NF-kappaB-independent and TRAF6-dependent mechanism. J Biol Chem.

[9] Kim MS, Zeng W, Yuan JP, Shin DM, Worley PF, Muallem S (2009). Native Store-operated Ca2+ Influx Requires the Channel Function of Orai1 and TRPC1. J Biol Chem.

[10] Hwang JK, Erkhembaatar M, Gu DR, Lee SH, Lee CH, Shin DM, Lee YR, Kim MS (2014). Glechoma hederacea Suppresses RANKL-mediated Osteoclastogenesis. J Dent Res.

[11] Park B, Yang YM, Choi BJ, Kim MS, Shin DM (2013). Activation of G Proteins by Aluminum Fluoride Enhances RANKL-Mediated Osteoclastogenesis. Korean J Physiol Pharma.

[12] Yang YM, Jung HH, Lee SJ, Choi HJ, Kim MS, Shin DM (2013). TRPM7 Is Essential for RANKL-Induced Osteoclastogenesis. Korean J Physiol Pharmacol.

[13] Yang YM, Kim MS, Son A, Hong JH, Kim KH, Seo JT, Lee SI, Shin DM (2009). Alteration of RANKL-induced osteoclastogenesis in primary cultured osteoclasts from SERCA2+/- mice. J Bone Min Res.

[14] Weidema AF, Dixon SJ, Sims SM (2001). Activation of P2Y but not P2X(4) nucleotide receptors causes elevation of [Ca2+]i in mammalian osteoclasts. Am J Physiol Cell Physiol.

[15] Korcok J, Raimundo LN, Ke HZ, Sims SM, Dixon SJ (2004). Extracellular nucleotides act through P2X7 receptors to activate NF-kappaB in osteoclasts. J Bone Min Res.

[16] Fuller K, Wong B, Fox S, Choi Y, Chambers TJ (1998). TRANCE is necessary and sufficient for osteoblast-mediated activation of bone resorption in osteoclasts. J Exp Med.

[17] Nunez G, Benedict MA, Hu Y, Inohara N (1998). Caspases: the proteases of the apoptotic pathway. Oncogene.

[18] Sugatani T, Alvarez U, Hruska KA (2003). PTEN regulates RANKL- and osteopontin-stimulated signal transduction during osteoclast differentiation and cell motility. J biol Chem.

[19] Miyamoto T (2011). Regulators of osteoclast differentiation and cell-cell fusion. Keio J Med.

